# Novel Isolating Approaches to Circulating Tumor Cell Enrichment Based on Microfluidics: A Review

**DOI:** 10.3390/mi15060706

**Published:** 2024-05-27

**Authors:** Zezheng Qiao, Xiangyu Teng, Anqin Liu, Wenguang Yang

**Affiliations:** 1 School of Electromechanical and Automotive Engineering, Yantai University, Yantai 264005, China; qiaojingbiao6656@163.com (Z.Q.); 15166828596@163.com (X.T.); 2 School of Mechanical and Electrical Engineering, Yantai Institute of Technology, Yantai 264005, China

**Keywords:** CTCs, microfluidic technologies, physical and biological properties, isolation methods

## Abstract

Circulating tumor cells (CTCs), derived from the primary tumor and carrying genetic information, contribute significantly to the process of tumor metastasis. The analysis and detection of CTCs can be used to assess the prognosis and treatment response in patients with tumors, as well as to help study the metastatic mechanisms of tumors and the development of new drugs. Since CTCs are very rare in the blood, it is a challenging problem to enrich CTCs efficiently. In this paper, we provide a comprehensive overview of microfluidics-based enrichment devices for CTCs in recent years. We explore in detail the methods of enrichment based on the physical or biological properties of CTCs; among them, physical properties cover factors such as size, density, and dielectric properties, while biological properties are mainly related to tumor-specific markers on the surface of CTCs. In addition, we provide an in-depth description of the methods for enrichment of single CTCs and illustrate the importance of single CTCs for performing tumor analyses. Future research will focus on aspects such as improving the separation efficiency, reducing costs, and increasing the detection sensitivity and accuracy.

## 1. Introduction

Metastasis of cancer is the main cause of patient death, and metastasis is a multi-step process [1]. Tumor cells are shed from the primary tumor, enter blood vessels or lymphatic vessels, and then are transported through the bloodstream to other tissues and organs in the body [2]. Finally, these cells adhere to the endothelium of the target tissue and then cross the endothelium into the target tissue to divide and proliferate, forming secondary tumors [3]. Most tumor cells that enter the bloodstream undergo apoptosis [4,5]. However, a minority of tumor cells are able to interact with the blood microenvironment during circulation and survive, which we call CTCs [6]. Programmed death ligand 1 (PD-L1) expressed on CTCs can help tumor cells evade detection and attack by immune cells [7,8]. In addition, the interaction of CTCs with platelets and macrophages can inhibit the anoikis phenomenon (a type of apoptosis caused by the loss of cellular attachment to the stroma) [9]. The interaction of neutrophils with CTCs generates chromatin webs called “neutrophil extracellular traps” (NETs) [10], which can facilitate the translocation of CTCs [11,12]. During metastasis, CTCs also undergo a mesenchymal–epithelial transition (MET) and an epithelial–mesenchymal transition (EMT), which alter the state of CTCs and promote their metastatic ability [13,14].

Recent studies have shown that CTCs have significant advantages as a liquid biopsy marker in the diagnosis, treatment, and prognosis assessment of cancer [15,16]. The collection of CTCs is very convenient, which can be completed by a simple blood sample collection, avoiding invasive and tissue injury during conventional surgical biopsy process [17]. Additionally, CTCs, as products of primary tumor circulating in the body, are more easily detected and have higher sensitivity compared to local tissue sampling. In addition, CTCs carry genetic information of the primary tumor and are highly representative [18]. Due to the heterogeneity of tumors, conventional biopsies may not be able to fully represent the characteristics of tumors. The analysis of CTCs can overcome this problem and provide more comprehensive and accurate information about the tumor, which can help to develop individual treatment plans [19]. Most importantly, the analysis of CTCs can also guide drug sensitivity detection and help doctors choose the most appropriate therapeutic drugs and dosages [20].

**Figure 1:**
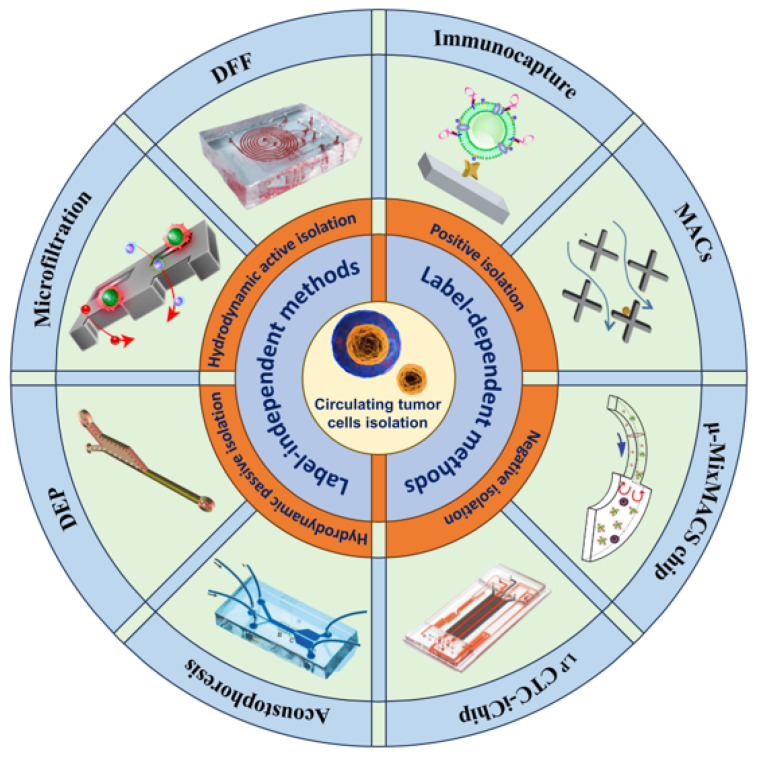
Schematic illustration of the method of isolation of CTCs.

In order to obtain accurate biopsy results for a liquid biopsy, a sufficient number of CTCs need to be enriched. However, CTCs are very rare in human blood, with less than 10 CTCs per milliliter of blood [21], so how to better enrich CTCs has become a hot research topic. Currently, the enrichment technologies for CTCs are mainly based on their biological and physical principles [22]. Label-dependent isolation methods based on biological principles make use of specific markers on the surface of CTCs to achieve enrichment, such as the epithelial cell adhesion molecule (EpCAM) [23,24], the human epidermal growth factor receptor-2 (HER2) [25,26], the estrogen receptor [27,28], and the prostate-specific membrane antigen [29]. Different markers have different significance, such as the fact that by combining liquid-like polymer chains and anti-EpCAM mechanisms, the non-specific binding of proteins and leukocyte pairs can be effectively attenuated, improving the capture efficiency of CTCs [30]. Label-independent isolation technologies based on physical principles have also been widely investigated for the enrichment of CTCs [31,32]. These technologies isolate CTCs based on differences in size, density, deformability, and dielectric properties (Figure 1).

In this paper, we describe in detail recent isolation technologies based on physical and biological principles of CTCs and discuss their advantages and limitations. In addition, multi-step isolation methods, negative selection methods based on biological principles, and single CTC isolation methods are also described in detail. Finally, we detail the clinical performance of CTCs in cancer and summarize the current status and application prospects of microfluidics in the field of CTCs isolation.

## 2. Label-Dependent Methods

Label-dependent methods are technologies that isolate and enrich CTCs based on differences in cell surface markers. The majority of the label-dependent methods use a positive enrichment strategy to enrich CTCs by specific cell surface markers, such as EGFR, EpCAM, MUC1, and HER2, as shown in Table 1. In addition to positive enrichment methods, there are negative enrichment methods that use leukocyte-specific surface markers (CD66b, CD45), to deplete white blood cells (WBCs) and thus obtain CTCs.

### 2.1. Immunocapture

Immunocapture is a conventional method of CTCs isolation, the basic principle of which is to capture CTCs by binding specific antigens on the surface of CTCs to each other with specific antibodies or aptamers [33]. In a microchannel, antibodies or aptamers are coated on the inner surface of the microchannel, and as the blood sample passes through, the CTCs will bind to the antibodies or aptamers and be captured in the microchannel, while other blood cells flow out. At present, in order to improve the efficiency of CTCs isolation, researchers have designed the structures inside a variety of microchannels using different materials.

#### 2.1.1. Aptamer-Based CTCs Isolation

**Figure 2:**
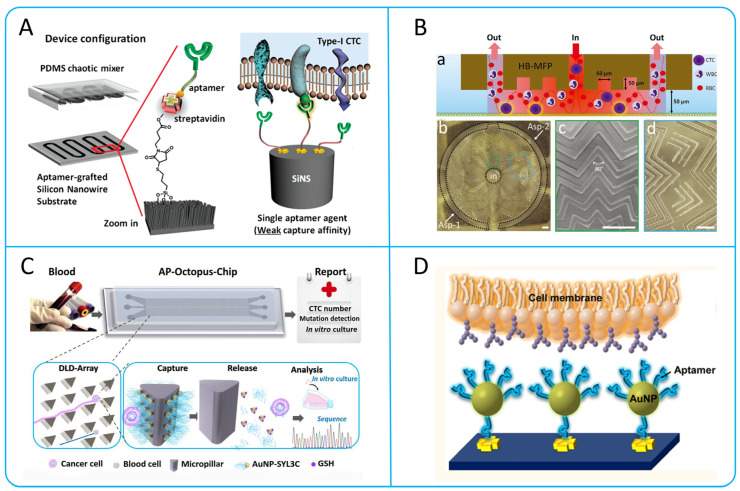
Typical immunocapture microfluidic device. (**A**) Using aptamer cocktail to enrich CTCs in patients with lung cancer; reproduced from Reference [35], with a permission from *Small*. (**B**) Herringbone microfluidic probe. (a) The schematic shows the working process of HB-MFP, in which blood is injected through the central aperture and exits through the peripheral aperture. (b) The tip surface of 3D-printed HB-MFP. (c,d) Specifically showing the surface structure in (b); reproduced from Reference [40], with permission from *Advanced Materials Technologies*. (**C**) Scheme shows the structural composition and working principle of the Ap Octopus Chip. The chip captures CTCs, using the synergistic manner of multivalent aptamers formed by AuNP-SYL3C and a triangular DLD array design. In the sequencing curves, green, red, blue and black represent adenine (A), thymine (T), cytosine (C) and guanine (G); respectively; reproduced from Reference [48], with permission from *Angewandte Chemie International Edition*. (**D**) The process of capturing CTCs using aptamer modified AuNP surfaces; reproduced from Reference [49], with permission from *ACS Nano*.

The use of aptamers has become widespread due to the limited number of CTC surface markers. Aptamers are chemical antibodies that can be manufactured artificially. Compared to antibodies, aptamers have high affinity, small size, and good stability. Zhang et al. developed a core-satellite-sized magnetic separable nanodevice (MS-RI), which recognizes intracellular nucleic acid targets and protein targets of CTCs by aptamers, thus enabling the capture and subtype identification of CTCs [34]. Zhao et al. developed a microfluidic chip with synergistic effects by embedding aptamer cocktails on SINS, as shown in Figure 2A. This system could differentiate and enhance the capture of various CTCs phenotypes from NSCLC patients through the synergistic effect with the aptamer panel, providing a higher capture efficiency [35].

#### 2.1.2. Antibody Functionalized Micropost Array

A micropost array covered in microchannels with antibodies specific for CTCs will change the flow line and increase the number of CTCs–microposts interactions, improving the efficiency of CTC capture. Nagrath et al. designed a microfluidic system (CTCs–Chip) that does not require pre-processing and labeling of samples, with 78,000 microposts covered with antibodies inside the microchannels to enhance interactions with the cell surface [36]. Ahmed et al. proposed a size-dictated immunocapture chip (SDI-Chip). The chip is equipped with a triangular array covered with specific antibodies, which can provide less shear stress and a higher interaction than a circular array. The capture efficiency of the CRC cells by the device was greater than 92%, and the cell purity was greater than 82% [37].

#### 2.1.3. Chaotic Mixing Microchannel

Chaotic mixing is also an effective way to increase surface contact between cells and functionalized antibodies or aptamers [38]. Wang et al. designed a wave-HB chip with wavy herringbone-micro-patterned surfaces, which eliminates the problem of high shear stress in the traditional HB-chip and improves the capture efficiency by up to 85% [39]. Glia et al. recently designed and successfully detected a novel microfluidic probe, the herringbone microfluidic probe (HB-MFP) as shown in Figure 2B. This probe uses radial staggered herringbone (HB) elements to increase cell capture efficiency by generating a microvortex. The research team successfully captured the CTCs cluster using prostate-specific antigens (PSA) [40].

#### 2.1.4. Nanomaterials with High Surface Area Ratio

In addition to changing the microchannel structure, nanomaterials and nanostructures with a high surface area ratio can also be used to increase the surface coated with antibodies [41]. For example, silicon nanopillars (SINP) [42], carbon nanotubes (CNTs) [43], silicon nanowires [44], graphene oxide (GO) [45], and nanofibers coated with antibodies [46] can improve the capture efficiency and purity of CTCs. In addition, magnetic nanoparticles combined with cell membranes have a higher ability to bind to extracellular epidermal growth factor receptor proteins, which can reduce non-specific adsorption of leukocytes and enhance the accuracy of identifying EGFR-positive CTCs [47]. Song et al. designed a DLD microfluidic chip (AP Octopus Chip) using aptamer functionalized nanospheres by simulating the multivalent pore interface of octopuses, as shown in Figure 2C. Compared with chips modified with monovalent aptamer, the multivalent aptamer-antigen binding can improve capture efficiency by up to 300%. In addition, CTCs can be released through a thiol exchange reaction without damaging them, with a release efficiency of up to 80% [48]. Sheng et al. combined multivalent DNA gold nanoparticles (AuNPs) with microfluidic devices and introduced herringbone-mixing microstructures to design an efficient capture platform, as shown in Figure 2D [49].

To sum up, the development and application of aptamers can overcome the problem of insufficient surface antigens of CTCs and improve the selectivity. Antibody-enabled micropost array and chaotic hybrid structures have high throughput. However, the micropost array imposes a high shear force on the CTCs, and the chaotic mixing structure is more difficult in the subsequent isolation of CTCs. Nanomaterials and nanostructures with high specific surface area not only increase the capture efficiency of CTCs, but also enable non-destructive release of CTCs.

### 2.2. Immunomagnetic Capture

**Figure 3:**
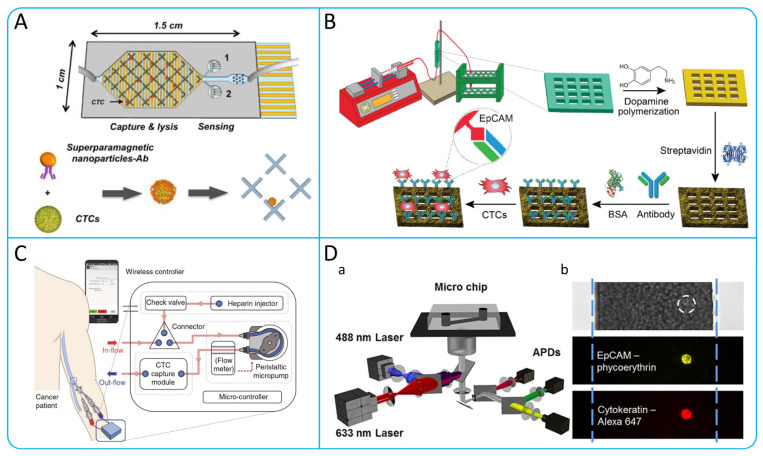
(**A**) Structural design and working principle display of VV chip; reproduced from Reference [53], with permission from *Analytical Chemistry*. (**B**) Schematic illustration of 3D-printed microfluidic device and immobilization procedures of anti-EpCAM antibody; reproduced from Reference [54], with a permission from *Biosensors and Bioelectronics*. (**C**) Schematic illustration of the indwelling intravascular aphaeretic system and its components functions; reproduced from Reference [57], with a permission from *Nature Communications*. (**D**) Schematic illustration of Microfluidic detection platform and cell imaging. (a) Description of the components of the detection platform. (b) Imaging of well-cultured MCF-7 cells was observed by a line-confocal microscope in microchannels filled with whole blood. The white dashed circle shows the location of the MCF-7 cell that is not visible beneath the many blood cells; reproduced from Reference [61], with permission from *Analytical Chemistry*.

The principle of the immunomagnetic capture method is to use magnetic nanoparticles or magnetic particles covered with antibodies and aptamers on the surface to bind to CTCs, which are then separated from blood cells by a magnetic field [50]. The Cell Search system is the first CTCs isolation system approved by the Food and Drug Administration (FDA) based on immunomagnetic capture technology [51]. Up to now, immunomagnetic capture methods have been more commonly studied. Qin et al. used polydopamine (PDA) coating and produced dynamic magnetic particles (DBMPs) using phenylboric acid (PBA) through the reversible catechol–borate interaction [52]. Mohamadi’s team combined the velocity valley chip with an integrated circuit, which has the ability to directly analyze the gene expression of CTCs in the manner shown in Figure 3A, avoiding the shortcomings of immunostaining methods that are slow to observe CTCs [53]. Chen et al. developed a unique microfluidic device using 3D printing technology, as shown in Figure 3B, which has a special internal structure with high surface area and can effectively control fluid flow. By using this device, the efficiency of capturing CTCs can reach up to 90% [54].

Recently, a number of research teams have worked to address the high-throughput continuity problems faced when applying immunomagnetic capture methods to enrich CTCs, and have designed innovative microfluidic systems, which can capture CTCs from peripheral blood directly [55]. Kefayat et al. designed a dialysis system for CTCs using a novel nuclear shell copper ferrite composed of Cu-CuFe2O4 and MIL-88A, and a 3D printed microfilter with large amounts of magnetic nanoparticles distributed. By applying a high-pressure magnetic field to the filter, large amounts of blood can be processed for a shorter time to isolate high viability CTCs and without affecting the viability of normal cells [56]. Kim et al. developed a temporary indwelling intravascular aphaeretic system for in vivo enrichment of CTCs, as shown in Figure 3C [57]. The system contains internal components such as a check valve, a peristaltic micropump, and a CTCs capture module. The microfluidic herringbone graphene oxide CTCs chip (HBGO chip) of the capture module captures CTCs before the blood flows back into the body. The system can continuously enrich CTCs, screening 1–2% of the entire bloodstream in 2 h. Those methods break through the limitations of traditional methods and provide a new way to capture CTCs efficiently.

To sum up, the immunomagnetic capture method, as a positive selection method, exhibits better selectivity and specificity compared to the negative selection method. Currently, the immunomagnetic capture method mainly relies on immunomagnetic particles, however, this approach leads to CTCs being adsorbed onto the particles, making subsequent analysis difficult. In addition to immunomagnetic particles, the use of 3D printing technology in microfluidic devices has facilitated the design of magnetic internal structures with high surface area ratios, thereby improving the capture efficiency of CTCs.

### 2.3. Immunofluorescence

Fluorescence in situ hybridization (FISH) is a technique that uses fluorescent labeled probes to locate and detect specific DNA sequences in cells or tissues [58]. The traditional fluorescence-activated cell sorter (FACS) is limited by its large size and the requirements of well-trained personnel [59]. Therefore, a microfluidic-based FISH device has been created for CTCs detection, offering significant time and cost savings over traditional FISH methods [60]. In Figure 3D, Zhao et al. developed a high-throughput CTCs counting method combining microfluidics and line-confocal microscopy, enabling the direct labeling of CTCs with fluorescent antibodies, rapid analysis of 1 mL blood samples in under 30 min, and achieving a 94% (n = 9) recovery rate in breast cancer cells [61].

**Table 1:** Label-dependent microfluidic methods for CTCs isolation.

Isolation Technologies	Working Principle	Specific Methods	Advantages	Limitations	Cancer Cell Lines	Throughput (mL h^−1^)	Recovery	Reference
Immunocapture	Chaotic mixing	HB-Chip	Microvortex can be generated to enhance the interaction of CTCs with the antibody-coated chip surface.	CTCs are hard to release.	PC-3	1	91.8% ± 5.2%	[62]
	Micropost array	OncoBean-Chip	Ultra-high-throughput processing of blood samples.	Relatively high production costs.	H1650, MCF-7	10	>80%	[63]
	Nanomaterial	Nanovesicles	Has the advantage of a natural biomembrane. Minimizes blood cell adsorption.	Low throughput.	MCF-7, HCT116, LNCaP	0.35	>70%	[64]
	Aptamer	AP-Octopus-Chip	Multivalent aptamers improve CTCs capture efficiency.	Complicated to manufacture. Low throughput.	SW480, LNCap	1	86.7%–89.4%	[48]
Immunomagnetic capture	Velocity valley	VV-Chip	Flow rate of blood samples can be controlled.	CTCs are hard to release.	DU145	2	97%	[53]
	Direct capture		High throughput. Convenient operation.	Collected CTCs have low activity.	COLO205	10	90%	[65]
	Magnetic filtration		Ultra-high-throughput enrichment of CTCs.	Magnetic beads on CTCs are difficult to remove.	MCF-7	120	89%	[66]
	Micromixer	μ-MixMACS	Increased WBCs depletion by generating micro-vortexes.	Lower purity of enriched CTCs.	MCF-7	18	>80%	[67]
	Vivo enrichment	Magnetic wire	Enrichment of CTCs directly from human whole blood.	Somewhat invasive to the human body.	nH1650, NSCLC	1200	49 ± 8%	[55]
Immunofluorescence	Immunostaining		High sensitivity, high specificity, and visualization.	False-positive or false-negative results may occur.	MCF-7	2	94%	[61]

## 3. Label-Independent Methods

There are many limitations in isolating CTCs from whole blood samples based on their biological properties. Some specific surface markers on CTCs are rarely or not expressed at all, such as epithelial cell adhesion molecules (EpCAM) and members of the cytokeratin family (CK8, CK18, and CK19). In particular, when CTCs undergo an EMT process, EpCAM are down-regulated, resulting in the loss of CTCs when collected using methods that rely on the immune properties of CTCs [68,69]. Current studies have found that CTCs and blood cells differ in size [70], deformability [71], dielectric properties [72], and other physical characteristics [73]. The isolation method based on the physical properties of CTCs has the characteristics of high throughput, low invasiveness, and repeatability, and the collected cells have high activity, which is convenient for downstream detection and analysis [74]. The typical isolation methods based on physical principles are summarized in Table 2.

### 3.1. Hydrodynamic Passive Isolation

#### 3.1.1. Dean Flow Fractionation (DFF) and Inertial Focusing

DFF and Inertial focusing isolation is a cell isolation method based on the principles of microchannel hydrodynamics. CTCs moving in curved microchannels can be isolated from blood samples by forces such as shear-induced lift force, wall-induced lift force, and Dean drag force [75].

##### Spiral Microchannel

**Figure 4:**
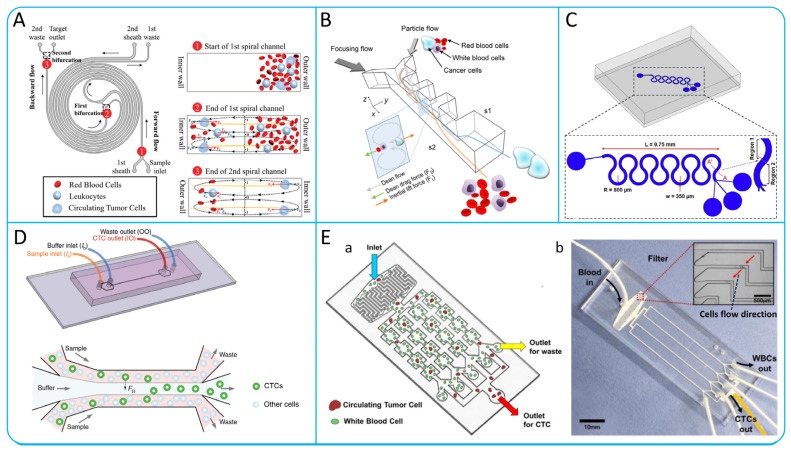
(**A**) Schematic illustration of the double spiral microchannel to achieve cell focusing. The diagram is labeled with the inlets, outlets, and the direction of flow. The numbers 1, 2, 3 represent the flow state of cells in three different positions of the microchannel; reproduced from Reference [76], with permission from *Frontiers in Bioengineering and Biotechnology*. (**B**) Schematic illustration of the working principle of the CEA microchannel; reproduced from Reference [78], with permission from *Analytical Chemistry*. (**C**) Isolation of CTCs by a serpentine microchannel; reproduced from Reference [17], with permission from *Micro and Nano Engineering*. (**D**) Schematic illustration of the microfluidic device and working principle of inertial migration in microfluidic channel; reproduced from Reference [83], with permission from *Microsystems & Nanoengineering*. (**E**) Schematic illustration of p-MOFF device. (a) Working principle diagram. (b) Microfiltration microscopy images; reproduced from Reference [79], with permission from *Biosensors and Bioelectronics*.

Spiral microchannel is known for its unique spiral shape and extremely small size, which can enhance the mixing efficiency between fluids and precisely control the fluid flow rate. In Figure 4A, Li et al. developed a spiral microchannel for high-purity CTCs isolation using Dean migration and inertial focusing technologies. Blood is pumped through the outer wall of the microchannel while a sheath fluid is introduced at the inner wall to guide the blood cells closer to the outer edge. Larger CTCs are subjected to stronger inertial force at the inner wall, leading to inertial focusing and isolation from smaller blood cells, which return to the outer wall along the Dean vortex [76]. In order to improve the isolation efficiency, Abdulla et al. designed a novel integrated two-stage helical chip consisting of two helical microchannels and a zig–zag channel. The design effectively isolated two types of tumor cells, A549 and MCF-7, with isolation efficiencies of 80.75% and 73.75%, respectively, but the CTCs purity was not improved [77].

##### Contraction−Expansion Array (CEA) Microchannel

CEA microchannel which is a contraction–expansion asymmetric microchannel as shown in Figure 4B [78]. Blood samples are injected into the microchannel from the S1 side and their velocity increases when passing through the constriction zone. Cells are affected by inertial lift force and Dean drag force, and different sizes have different equilibrium positions. Larger cancer cells migrate towards S1, while smaller blood cells migrate towards S2. The device successfully achieved a 99.1% CTCs recovery and an 88.9% blood cell rejection. The P-MOFF device is a parallel porous flow sorting device that arranges a series of symmetrical contraction–expansion microchannels on a single chip, as shown in Figure 4E [79]. The main key to this method is to use the inertial lift caused by the change in momentum [80]. When the Reynolds number of the particles reaches 70, human breast cancer cells can be successfully isolated [81]. Compared to single-well flow sorting, P-MOFF has the advantage of higher isolation efficiency and high throughput, and can isolate CTCs directly from human peripheral blood without the need for dilution treatment [79].

##### Serpentine Microchannel

The serpentine microchannel is different from the previously described microchannel isolation mechanism. It exclusively utilizes Dean drag and centrifugal force, without relying on inertial lift. The serpentine microchannel is a microchannel with a certain curvature as shown in Figure 4C [17]. Dean vortexes are created when fluids flow in curved microchannels, causing cells in the microfluid to be subject to viscous drag, also known as Dean drag force [76]. In addition, the cells are subjected to centrifugal forces generated by inertia at the curves. Due to the interaction of centrifugal force and Dean drag force, the cell focuses at the center of the microchannel after multiple cycles of deflection. Zhang et al. analyzed for the first time the focusing pattern of different particles in the serpentine microchannel according to different conditions. They performed the isolation of polystyrene mixtures under high-throughput conditions (600 μm/min) and obtained isolation purities of more than 90% and more than 99% [82]. Mehrdad et al. studied the inertial focusing behaviors of different tumor cell lines MDA-MB-231 (11–22 μm), Jurkat (8–17 μm), K562 (8–22 μm), and HeLa (16–29 μm) in curved microchannels with a curvature angle of 280°. The results show that as the curvature angle increases, the Dean drag force increases [17]. The serpentine microchannel not only has the advantages of high throughput, simple operation, and reproducible utilization, but also has good parallelism and a small footprint compared to the spiral microchannel [82].

##### Straight Microchannel

In contrast to spiral and contraction–expansion microchannel, the straight microchannel uses only inertial lift force to focus cells to a certain position without the involvement of Dean drag force [83]. In a low-aspect-ratio microchannel, cells will migrate to the top and bottom of the channel under shear-induced lift force and wall-induced lift force, and then will move to the center of the top and bottom under rotation-induced lift force. Zhou et al. developed a novel MFM device using the focusing principle of straight microchannels, as shown in Figure 4D. The device confines the blood sample to the two sides of the microchannel and uses the rotation-induced lift force to move the CTC towards the center of the microchannel. In clinical application, the system successfully detected sex out of eight patients with non-small cell lung cancer (NSCLC) and achieved high isolation efficiency (>99%) and purity (83%) [83]. Straight microchannels have a simple channel design that does not require precise design of the radius of curve, channel length, etc.

#### 3.1.2. Microvortex

**Figure 5:**
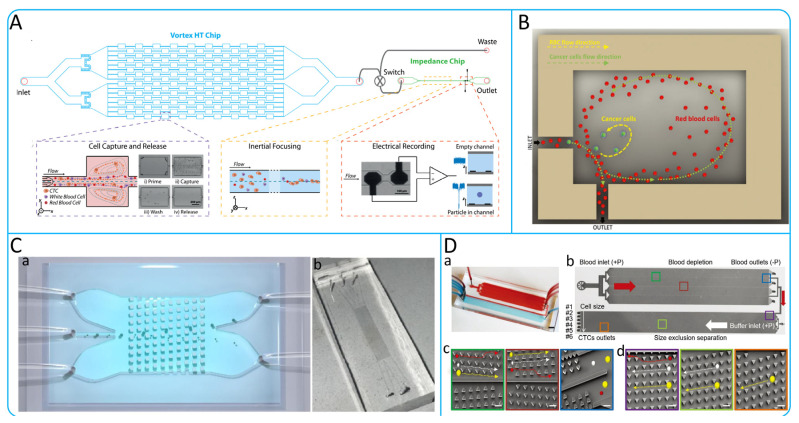
(**A**) Schematic illustration of the working principle of integrated microfluidic device. The device consists of two parts. The Vortex HT Chip can achieve high-throughput enrichment of CTCs, and the impedance chip can count the collected CTCs; reproduced from Reference [90], with permission from *Cytometry Part A*. (**B**) Schematic illustration of the working principle of the orthogonal vortex chip. Schematic diagram of the working principle of the vortex chip with orthogonal reversal. Enrichment of CTCs by using the generated orthogonal vortex and Dean drag force; reproduced from Reference [86], with permission from *Analytica Chimica Acta*. (**C**) TO DLD Chip Device Isolation CTCs. (a) The schematic illustration of the wide TO DLD chip. (b) The PDMS microchannel in TO DLD chip; reproduced from Reference [94], with permission from the *Journal of Chromatography A*. (**D**) Integrated Microfluidic Chip for isolation of CTCs. (a) Schematic illustration of the microfluidic chip. (b) Working schematic diagram for separating CTCs according to size inside a microchannel. (c,d) Triangular DLD arrays within the microfluidic channel for separating cells of different sizes. Cancer cells (yellow), red blood cells (red), and white blood cells (white); reproduced from Reference [95], with permission from *Advanced Biosystems*.

Inertial microfluids in a state of uniform flow can be observed in a separating Stokes flow phenomenon, known as Moffat eddies, in the presence of a sudden expansion of the microchannel [84,85]. As the flow Reynolds number increases, the Moffat eddies fully develop into microscale vortexes [86]. Wang et al. designed a simple vortex microfluidic device with high efficiency (90%) and purity (90%) for the isolation of polystyrene particles smaller than 2 μm under high-throughput conditions [87]. Sollier et al. invented a Vortex chip with eight parallel high aspect ratio microchannels, each with 8 chambers for enrichment of CTCs [88]. Che et al. designed a high-throughput vortex chip (Vortex HT Chip) for label-free, size-based enrichment of CTCs. Compared to the Vortex Chip, the Vortex HT Chip was optimized in terms of parallel processing power, with a 1.6-fold increase in isolation efficiency, purity up to more than 80%, and high viability of collected CTCs [89]. To simultaneously achieve enrichment and detection of CTCs. Raillon et al. combined the Vortex HT Chip and the Impedance Chip to automate the isolation and counting of CTCs, as shown in Figure 5A. Cells were collected using the Vortex HT Chip and released by decreasing the velocity, and then counted in different sizes according to the electric field fluctuation properties of the Impedance Chip [90]. To avoid larger shear stress at high flow rates leading to destruction of CTCs and difficulties in downstream detection, Rastogi et al. designed an orthogonal vortex chip as shown in Figure 5B [86]. The chip consists of a series of entry-exit microchannels orthogonally coupled to the capture chamber. The orthogonal arrangement allows vortex formation at low flow rates. The turn-effect is utilized thereby allowing larger cells to be captured in the chambers and smaller cells to exit with the fluid flow. The reliability of the system was verified by the successful isolation of three malignant breast cancer cell lines (MDA-MB-231, MCF-7 and BT-549) [86].

#### 3.1.3. Deterministic Lateral Displacement

Deterministic lateral displacement (DLD) is a particle size-based sorting method in microfluidic channels which has excellent selectivity for various particle sizes and can effectively control the critical particle sizes. The isolation of CTCs using DLD is essentially due to the difference in size, and deformability of CTCs and blood cells [91]. DLD is achieved by designing arrays of microposts with a certain angular arrangement within the microfluidic channel. That is, there is a certain lateral distance between the previous column and the next one. The CTCs continuously undergo lateral movement through constant bumping against the micropost array thus achieving isolation. Loutherback et al. designed a mirrored triangular posts array that can isolate breast cancer cells (MDA-MB-231) at 10 mL/min, and capture efficiencies of up to 86% can be achieved [91]. Bhattacharjee et al. utilized COMSOL Multiphysics 5.4 software for the simulation and analysis of microchannels, achieving efficient isolation of CTCs (13.5 μm) and WBCs (6 μm) with efficiencies of 84% and 96%, respectively [92]. Further innovating, they applied an electrical network analogy to adjust fluidic resistance, successfully isolating lung (22.5 μm), prostate (10.64 μm), and breast (13.1 μm) cancer CTCs from WBCs (12 μm), with over 90% efficiency in all cases [93]. Tang et al. proposed a novel DLD chip (Wide TO DLD chip) based on an improved zig–zag pattern and topology optimization algorithm, as shown in Figure 5C. The pillar shape calculated by the topology optimization (TO) algorithm can better increase the collision rate between cells and microposts [94]. Liu et al. designed an integrated cell isolation device including two mirrored DLD arrays and a DLD array with increased angle as shown in Figure 5D. Experimental results on samples of breast, liver, and lung cancers showed that the enrichment of CTCs exceeded 90% at a throughput of 12 mL h^−1^ with a purity higher than 50% [95].

Micropost shapes of DLD can be triangular [95,96], cylindrical [92,93,94], rectangular [97,98], airfoil pillars [99], etc. When the cells within the microfluidic channel bump against the microposts, the larger cells will move from the original streamline to the new streamline, while the smaller cells will flow in the original streamline. Currently, cylindrical and triangular arrays are the more common choices, with triangular arrays reducing the shear force on CTCs compared to cylindrical arrays. Topology optimization algorithms currently available can be used to design better shaped columns to separate CTCs more efficiently.

### 3.2. Microfiltration

**Figure 6:**
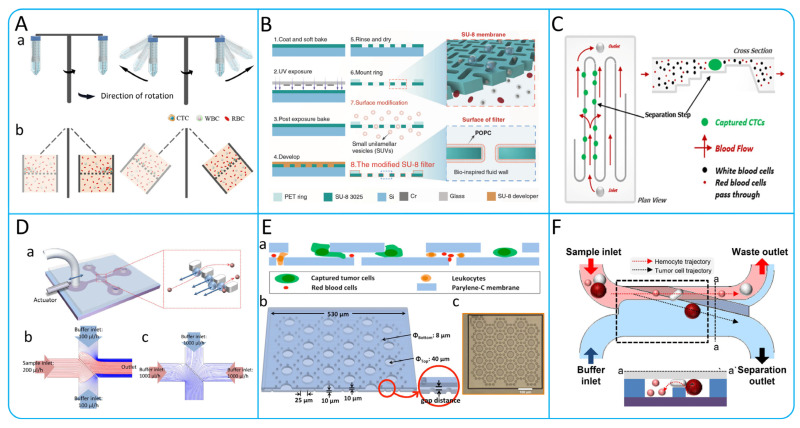
(**A**) Working principles of the filtration device. (a) Schematic illustration of centrifugal process. (b) Schematic illustration of cells in a blood sample passing through a cell uniformly distributed membrane during centrifugation; reproduced from Reference [101], with permission from *ACS Omega*. (**B**) Integrated microfluidic device with windmill-like hole array; reproduced from Reference [102], with permission from *Microsystems & Nanoengineering*. (**C**) Schematic illustration of the isolation principle of the Parsortix system; reproduced from Reference [104], with permission from *PLOS ONE*. (**D**) Illustration of the μ-sieving device. (a) The microfluidic device has four channels, with a piezoelectric actuator attached to the inlet channel. The red box shows the cell filtration process. (b,c) Schematic illustration of streamlines distribution in the sorting process; reproduced from Reference [105], with permission from *Scientific Reports*. (**E**) Design of separable bilayer microfiltration device. (a) Schematic illustration of the device cross-section. (b) 3D view of the device with geometric parameters. (c) Microscopic picture of the device. Scale bar is 100 μm; reproduced from Reference [103], with permission from *Scientific Reports*. (**F**) Schematic illustrations of the working principle of a slanted weir microfluidic device; reproduced from Reference [108], with permission from *Cancers*.

Microfiltration isolates CTCs based on size and deformability, retaining large CTCs while removing other blood components (RBCs, WBCs). Yusa et al. designed a 3D palladium filter achieving 90% recovery at 2.0–2.5 mL/min flow rate [100]. However, microfilters that isolate cells based on their size tend to cause cell clogging, reducing cell recovery and purity. To solve this problem, Han et al. designed a membrane filtration device with a horizontal rotor for even cell distribution, improving cells isolation in centrifuges, as shown in Figure 6A [101]. Li et al. designed a windmill-like uniform touch (SU-8 film) pore array, as shown in Figure 6B. The fluid above the micropores can be disturbed to induce the cells in the fluid to self-mix, so that the cells in the fluid can be evenly distributed to prevent blockage. Clinical evaluations showed high recovery rates of 93% for A549 cells and 90% for HeLa cells [102]. Taking into account the difficulty in releasing the collected CTCs, Zhou et al. developed a separable bilayer microfilter (Figure 6E) with a bilayer of biocompatible polymer parylene C for milder CTCs capture and release, reducing stress by designing 40 μm and 8 μm pores, with efficiencies of 83 ± 3% and 78 ± 4% for MCF-7 and MDA-MB-231 cells, respectively [103]. Compared to the pore structures, weir structures are widely used in microfilters due to their simple design and low cost. Weir structures utilize fixed physical barriers to achieve interception and isolation of particles in the fluid and rely on precise size control to match the size of the target particles. One of the most popular is the Parsortix system, as shown in Figure 6C [104]. By comparing Parsortix system with IsoFlux, the purity of CTCs obtained by Parsortix was 3.1%, which was significantly higher than that obtained by IsoFlux (1.0%) (*p* = 0.02).

Achieving continuous isolation of CTCs is one of the important characteristics of microfiltration devices. Although a variety of microfiltration devices exist on the market today that can alleviate the clogging problem to some extent, they still fail to achieve a constant and continuous enrichment of CTCs. To address this challenge, Yoon et al. developed a transversal flow microfluidic screening (μ-sieving) device (Figure 6D) that reduces cells clogging by adding low frequency oscillations to the fluid [105]. A piezoelectric actuator is attached to the left microchannel, which creates an oscillation that moves the sample solution back and forth in a small area. In contrast to previous studies [106,107] where filters were vibrated to reduce clogging, the oscillation of the fluid reduces the mechanical stress between the different components within the device, thus maintaining a stable fluid environment. The device can reach 100% efficiency and purity [105]. In addition, Yoon et al. developed an oblique weir microfluidic device, as shown in Figure 6F. The isolation of a breast cancer cell line (LM2 MDA-MB-231) confirmed the reliability of the device, which can achieve a high isolation efficiency of 97% with minimal blood cell contamination [108].

To sum up, achieving continuous isolation of CTCs and avoiding microfilter clogging is one of the main focuses of current research. Although pore and micropost structures can also avoid clogging, they require specific equipment and are costly. Compared to the pore and micropost structures, the weir structure is simpler, less prone to clogging, and allows for continuous CTCs isolation, but with lower purity of isolation.

### 3.3. Hydrodynamic Active Isolation

#### 3.3.1. Acoustophoresis

**Figure 7:**
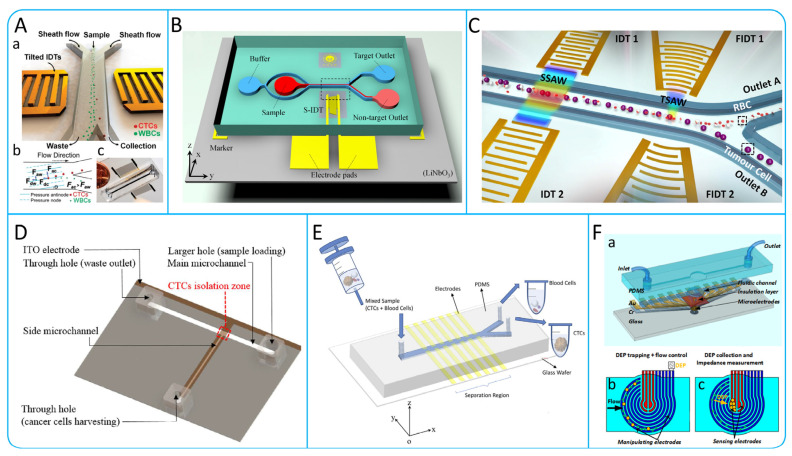
(**A**) The high-throughput taSSAW device for CTCs isolation. (a) Illustration of cell isolation in the taSSAW device. (b) Schematic illustration of the principle of taSSAW-based cell isolation. (c) Actual size image of the tasSAW cell isolation device; reproduced from Reference [110], with permission from the *Proceedings of the National Academy of Sciences*. (**B**) Schematic illustration of an ultra-compact acoustofluidic device based on np-TSAW for enrichment of CTCs; reproduced from Reference [111], with permission from *Analytica Chimica Acta*. (**C**) Schematic illustration of CTCs isolation based on multi-stage surface acoustic waves; reproduced from Reference [113], with permission from *Sensors and Actuators B: Chemical*. (**D**) Microfluidic system based on optically induced dielectrophoretic (ODEP); reproduced from Reference [115], with permission from *Scientific Reports*. (**E**) Schematic illustration of a microdevice based on LFFF-DEP; reproduced from Reference [116], with permission from the *Journal of Chromatography B*. (**F**) Microfluidic device for impedance detection and dielectrophoresis integration. (a) Structural diagram of the device. (b) Cell Flow and DEP trapping. (c) Identification of target cells by impedance; reproduced from Reference [117], with permission from *Biosensors and Bioelectronics*.

Acoustophoresis is a simple method of cells control that allows the isolation of CTCs by varying the amount of acoustic force applied to the cells depending on their density, deformation and size [109]. Li et al. designed a unique tilt-angle standing surface acoustic wave (taSSAW) structure shown in Figure 7A [110]. This structure utilizes interdigital transducers (IDTs) arranged at a specific angle to generate surface acoustic waves (SAWs), which interfere to form taSSAW and create parallel pressure nodes in the microfluidic channel. By detecting the drag force (Fd) and acoustic radiation force (Fr), the flow state of cells can be predicted. For larger cells, the acoustic radiation force dominates and leads to larger lateral displacements. The recovery of this device was greater than 83% for both breast-like cancer cells (MCF-7) and LNCaP cells. In order to improve the purity of CTCs, Geng et al. designed a device (Figure 7B) using a narrow-path traveling surface acoustic wave (np-TSAW) for efficient CTCs isolation, achieving over 98% efficiency and up to 93% purity with MCF-7 cells. Featuring a focused SAW beam and an open-circuit reflector that reduces the acoustic aperture to around 600 μm, this compact device (<2 × 1.5 cm^2^) offers practical applications in CTCs sorting [111].

At present, most SAW-based sorting tasks require the introduction of sheaths into pre-aligned samples via stream-focusing methods [112]. However, introducing the sheath into the flow will further dilute the rare CTCs. Wang et al. created a two-stage acoustic microfluidic device for CTCs isolation from blood, using surface acoustic waves (SAW), as shown in Figure 7C. The device features straight and focused interdigital transducers (IDTs and FIDTs) for generating standing SAWs (SSAWs) and focused traveling pulsed SAWs (TSAWs), respectively. This design efficiently isolates CTCs from red blood cells (RBCs). Detected with polystyrene particles and glioma cells, it achieved up to 94.2% isolation efficiency for 5 μm particles and 90% ± 2.4% for U87 glioma cells at 720k cycles, demonstrating its effectiveness in cell sorting [113].

#### 3.3.2. Dielectrophoresis

Dielectrophoresis is a technique used for isolating and analyzing biomolecules based on their different dielectric properties. When cells are placed in a non-uniform electric field, they experience varying electrophoretic forces, causing them to move. The cell membrane accumulates surface charge in the presence of an alternating electric field, known as membrane charging. At high frequencies, if the cell’s internal conductivity is higher than the surrounding medium, it experiences a positive dielectrophoretic force (positive FDEP). Conversely, at low frequencies, the cell’s exterior becomes charged and repels the electric field, resulting in negative dielectrophoretic forces (negative FDEP) [72]. Shim et al. developed a DEP-FFF system that can batch process blood samples and is able to achieve a processing efficiency of 106 nucleated cells/min [114]. In order to improve the purity of CTCs, Huang et al. designed a microfluidic system as shown in Figure 7D. This system combines fluorescence imaging technology and optically induced dielectrophoresis (ODEP) technology on the basis of traditional isolation methods to further improve the purification of CTCS. By detecting PC-3 cells, the results showed that the method was able to achieve a 100% purity collection of cancer cells [115]. Waheed et al. designed a system called “Lateral fluid flow fractionation (LFFF-DEP)” using two sets of planar interleaved sensor electrodes. In this system, they successfully isolated breast cancer cells (MDA-MB-231) labeled with green fluorescent protein (GFP) from blood, as shown in Figure 7E [116]. To better observe the isolation of CTCs, Nguyen et al. designed a device integrating electrophoresis and impedance detection, as shown in Figure 7F [117]. In this device, lung cancer cells (A549) would move towards the center of the working area under positive dielectrophoretic force and eventually be captured on the sensing electrodes. By detecting the change in impedance, the presence of the cells can be determined [117].

**Table 2:** Label-independent microfluidic methods for CTCs isolation.

Isolation Technologies	Working Principle	Specific Methods	Advantages	Limitations	Cancer Cell Lines	Throughput (mL h^−1^)	Recovery	Reference
Hydrodynamics	Inertial focusing	DFF	Continuous collection, high throughput.	Need to dilute blood samples.	MCF-7	3	85%	[75]
		CEA	Continuous high-throughput collection of CTCs under low shear stress.	Cell viability is greatly affected by flow velocity. Low purity.	MCF7, SK-BR-3	6	99.1%	[78]
		P-MOFF	Excellent parallelizability, high throughput.	Need to dilute blood samples, which may cause loss of tumor cells.	MCF-7	36	91.60%	[79]
		Symmetrical serpentine microchannel	Good parallelizability and portability.	Easy to lose smaller CTCs.	MEL	36	94.90%	[82]
		MFM	Without the need for complex sample preparation steps.	Higher shear stress.	NSCLC	18	>93%	[83]
	Deterministic lateral displacement	Two-stage DLD strategy	Can be integrated online with other CTCS analysis or capture technologies.	This method has lower throughput and lower efficiency in processing samples.	MCF-7	>0.25	99%	[118]
		Triangular Column Mirror Array	Ultra-high-throughput isolation of highly active CTCs.	CTCS clusters are hard to release.	MDA-MB-231, MCF10A	600	>85%	[91]
	Microfluidic Vortex	Vortex Chip	Enrichment of active CTCs in a continuous, high-throughput manner.	Low isolation efficiency.	MCF-7, PC-3, A549	24	20.7%	[88]
		Vortex HT	Good parallelizability, high throughput.	Higher shear stress.	MCF-7	48	>85%	[89]
Mechanical filtering	Size and deformation	CTCS cluster-chip	No pre-processing of blood samples required.	Lower sensitivity.	MCF-7	2.5	>80%	[119]
		Parsortix	Simple to operate with low cost	CTCs exhibit heterogeneity in size.	DU145	4	57.3% ± 8.3%	[104]
		MCA	Simple microchannel structure and convenient operation.	It is inevitable to lose smaller tumor cells and may cause blockage.	NCI-H820, A549	2.5	>70%	[70]
		Slanted Weir	Continuous isolation of CTCs without blockage.	Accurate analysis of the inclination angle of the weir is required.	LM2, MDA-MB-231	2.5–3.8	97%	[108]
		Microfluidic ratchet mechanism	CTCs captured with 25 times more than traditional Cell Search systems.	Relatively low throughput.	UM-UC-13	1	>90%	[71]
		3D Palladium Filter	Can withstand high pressures.	Pores fusion and low-density distribution can reduce capture efficiency.	NCI N-87, COLM-5	120–150	>85%	[100]
Dielectrophoresis	DEP	DEP-FFF	Continuous processing of whole blood cells with high sensitivity.	The dielectric properties may change due to treatment plan.	MDA-MB-231	1.2	70%–80%	[114]
		ODEP	This system can achieve up to 100% purity of CTCs.	Low throughput and low recovery.	PC-3	0.15	41.5%	[115]
		DEP-MOFF	Can continuously, quickly, and high-purity isolate CTCs.	During the isolation process, the system can cause cell loss.	MCF-7	7.5	>75.18%	[120]
Acoustophoresis	Aoustic radiation force	Acoustophoresis Chip	Label-free isolation with high sensitivity.	Low throughput and lack of long-term stability of equipment.	DU145	4.2	>83.7%	[109]
		taSSAW	Excellent biocompatibility, simple design, and label free automated operation.	Any instability of the pressure source may lead to deviations.	MCF-7, UACC903	1.2	>83%	[110]
		Hybrid PDMS–glass microchannel	Maintaining the integrity, function and viability of CTCs.	The equipment process is complex, and the cost is high.	PC-3, LnCaP	7.5	>86%	[112]

## 4. Multi-Step CTCs Isolation Methods

### 4.1. Multi-Step Isolation Based on Physical Principles

**Figure 8:**
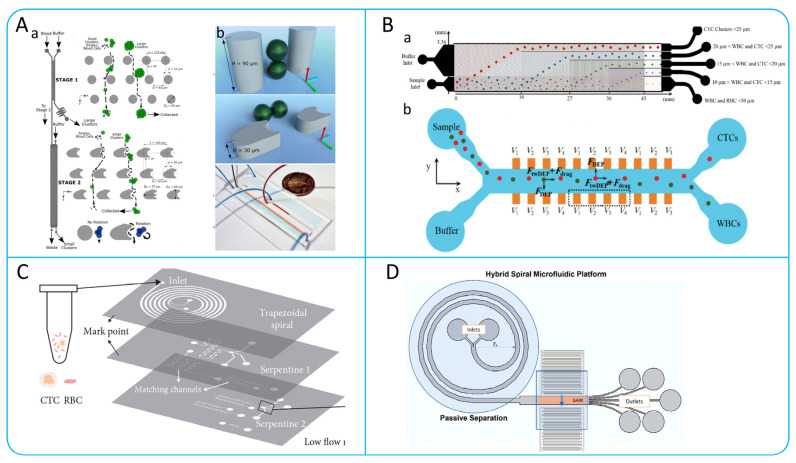
(**A**) DLD-based two-stage continuous cells isolation device. (a) Schematic diagram of the device for cells isolation. (b) Specific ways in which cells pass through micropost arrays; reproduced from Reference [118], with permission from *Scientific Reports*. (**B**) Microfluidic devices based on DLD and DEP integration. (a) Schematic illustration of cell isolation based on DLD arrays. (b) Schematic diagram of the working principle of the dep device; reproduced from Reference [123], with permission from the *Journal of Molecular Liquids*. (**C**) A 3D-Stacked Multistage Inertial Microfluidic Chip; reproduced from Reference [124], with a permission from *Cyborg and Bionic Systems*. (**D**) A hybrid helical microfluidic platform coupled with surface acoustic waves; reproduced from Reference [125], with permission from *Biosensors*.

In order to better rapidly collect high-purity CTCs, many researchers have developed a series of multi-step CTCs isolation methods based on microfluidic systems. For example, the multi-stage application of a principle, or the combination of various principles and technologies to improve the collection efficiency and purity of CTCs [121,122]. Many current CTCs isolation devices cannot accomplish the isolation of CTCs clusters from a single cell [75]. In order to address this issue, Au et al. developed a two-stage microfluidic chip-based isolation system as shown in Figure 8A. The system is internally designed with deterministic lateral displacement arrays with different shapes, where the first stage uses cylindrical arrays to isolate large clusters of CTCs from blood cells, while the second stage deflects small clusters through asymmetric column arrays to achieve further isolation of CTCs. The experimental results of this system on breast cancer cell clusters show that the isolation efficiency is greater than 90%, and the viabilities of isolated cells is greater than 87% [118].

To improve the purity of CTCs isolation, many microfluidic platforms combining multiple principles have been designed. A hybrid microfluidic device for label-free CTCs isolation was presented by Varmazyari et al. [123]. This device consists of a DLD array and a twDEP system as shown in Figure 8B, which is capable of efficiently sorting cells with diameters in the range of 10–25 μm and enabling further isolation. Experimental results showed that the device was 93% efficient in recovering MDA-MB-231 cells from blood samples [123]. Xu et al. proposed a 3D-stacked multistage inertial microfluidic chip, as shown in Figure 8C. The device can isolate tumor cells (SW480, A549, and KAKI-1) from massive RBCs at a high inlet flow rate of 1.3 mL/min with isolation efficiency >80% and purity >90% [124]. Altay et al. combined helical microchannels with acoustic principles to design a hybrid helical microfluidic platform as shown in Figure 8D, enabling efficient isolation of CTCs [125].

### 4.2. Multi-Step Isolation Based on Biological and Physical Principles

**Figure 9:**
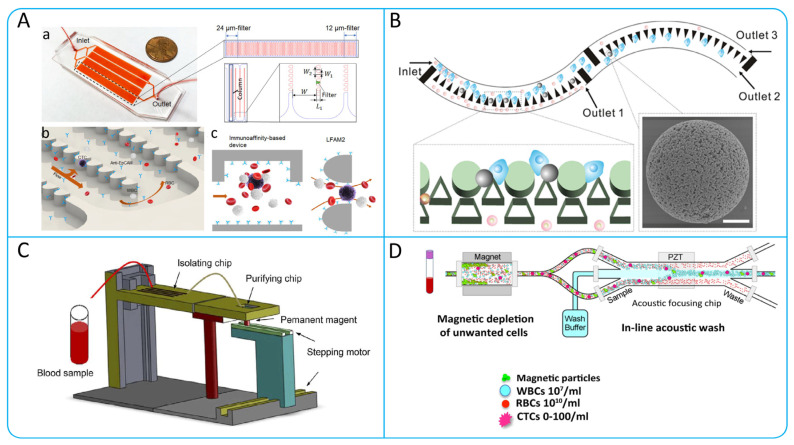
(**A**) Efficient isolation device based on lateral microfiltration combined with immunoaffinity. (a) Photo of the LFAM2 device. (b) Schematic diagram of the internal structure of the device. (c) Illustration of capturing cells; reproduced from Reference [127], with permission from *Scientific Reports*. (**B**) Filter chip with inertial microfluidics and antibody-functional microspheres; reproduced from Reference [128], with permission from *ACS Applied Materials & Interfaces*. (**C**) Integrated cell isolation device based on DLD arrays and immunomagnetic beads; reproduced from Reference [129], with permission from *Scientific Reports*. (**D**) Schematic illustration of the working principle of the integrated chip based on immunomagnetic isolation and acoustic isolation; reproduced from Reference [130], with permission from *Scientific Reports*.

Researchers have developed a number of microfluidic systems that combine physical and biological properties. These microfluidic systems not only leverage the high-throughput advantages of physical isolation methods for processing large sample numbers but also exploit the high purity offered by immunoaffinity technologies [126]. In the study by Chen et al. they designed a microfluidic system for the capture of CTCs using immunoaffinity and lateral microfiltration technologies as shown in Figure 9A. The system consists of four serpentine microchannels, each containing a series of lateral filters with a size range of 24–12 μm, similar to CTCs. These filters bind to specific antibodies and are able to selectively capture CTCs, and the capture efficiency of the system can be as high as between 87.2% and 93.5% [127]. Su et al. developed an antibody-functional microsphere-integrated filter chip, as shown in Figure 9B, which uses circular channels with triangular arrays and cylindrical arrays and is covered with microspheres with nanostructures [128]. The design of this chip enables it to efficiently capture different cancer cell lines with capture efficiencies of more than 90%. Similarly, Jiang et al. designed an integrated microfluidic device for the rapid and highly sensitive detection of CTCs as shown in Figure 9C. The device consists of three components: a DLD array for isolating CTCs, CD45-labeled immunomagnetic beads for removing WBCs, and a capture platform coated with rat tail collagen. The device captures green fluorescent protein (GFP)-positive cells in blood with capture efficiencies of up to about 90% and is capable of achieving purity of about 50% [129]. Usually, molecular analysis of CTCs and cell clusters is susceptible to low purity and cell loss. Bhagwat et al. designed an integrated platform based on the principles of flow cytometry as shown in Figure 9D [130]. The device first depletes the WBCs count of a blood sample by immunomagnetic capture, and then the acoustophoresis is used to further improve the purity of CTCs. In addition, given that CTCs become more invasive after undergoing an EMT process, Topa et al. designed a device based on negative anti-CD45 enrichment, density gradient centrifugation, and multiplexed immunofluorescence staining to isolate CTCs undergoing an EMT process [131].

To sum up, isolation methods based on a combination of physical and biological properties of CTCs have been widely used. These methods not only have the advantage of high throughput, but also make full use of biological principles to improve the sensitivity to CTCs, thus achieving efficient collection of high-purity CTCs. Among these methods, microfluidic devices combining immunomagnetic beads and microfiltration are the most widely used. In addition, methods based on the combination of acoustophoresis and immunomagnetic particles have shown the potential to isolate CTCs efficiently and without loss. The continuous development of these techniques provides more options for the isolation of CTCs and provides important support for further research and clinical applications.

## 5. Negative Enrichment of CTCs

**Figure 10:**
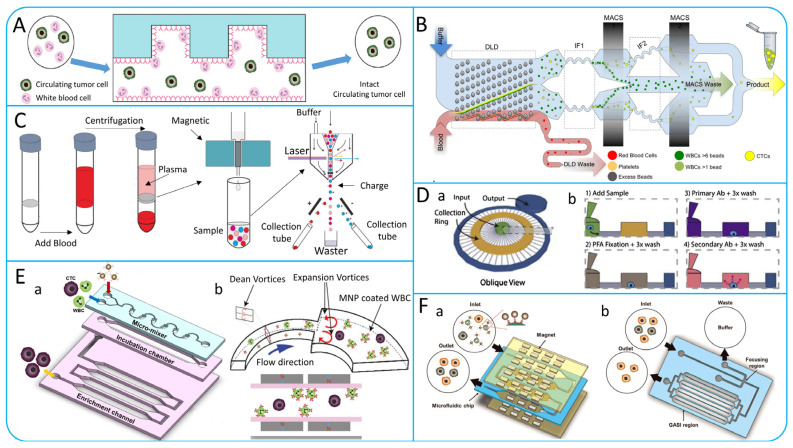
(**A**) Negative enrichment of CTCs based on GASI-ship; reproduced from Reference [132], with permission from *Analytical Chemistry*. (**B**) Monolithic chip for isolation of CTCs; reproduced from Reference [134], with permission from *Scientific Reports*. (**C**) Immunomagnetic negative enrichment coupled with flow cytometry to isolate CTCs; reproduced from Reference [135], with permission from *Cancer*. (**D**) Microfluidic Cell Concentrator for Negative Enrichment of CTCs. (a) 3D schematic diagram of the device. (b) CTCs collection process; reproduced from Reference [136], with permission from *Methods*. (**E**) μ-MixMACS chip. (a) Schematic illustration of the structure of the chip. (b) Illustration of the working principle of this chip to capture CTCs; reproduced from Reference [67], with permission from *Sensors and Actuators B: Chemical*. (**F**) Two-stage microfluidic chip. (a) Leukocyte-depleted μ-MACS chips. (b) Illustration of GASI chip; reproduced from Reference [133], with permission from *Biosensors and Bioelectronics*.

Positive selection methods mainly rely on the expression of specific antigens on the surface of CTCs. However, during CTCs metastasis, CTCs may undergo EMT [13], which results in a decrease in the expression of specific antigens, thus affecting the accuracy of the assay. In addition, CTCs modified by specific antibodies may interfere with downstream molecular analysis. As a result, negative selection methods have been extensively studied. Hyun et al. proposed a microfluidic chip called Geometrically Activated Surface Interaction Chip (GASI-chip), the structure of which is shown in Figure 10A [132]. The chip generates mixing flows through a herringbone structure, which enhances the interaction of nontarget cells with the microchannel surface. They then combined a microfluidic magnetic-activated cell sorter (μ-MACS) with the GASI-chip to design a two-stage microfluidic chip as shown in Figure 10F [133], which can process 400 μL/min of blood samples. In addition, Fachin et al. designed a high-throughput, automated microfluidic chip containing 128 deterministic lateral displacement (DLD) devices for RBCs removal and two cascaded MACS devices for WBCs removal [134]. As shown in Figure 10B, the chip has the capacity to process 15–20 million cells per second. In Figure 10C, Lu et al. developed a method combining immunomagnetic negative enrichment and fluorescence-activated cell sorting for the detection and isolation of CTCs with high sensitivity and recovery [135]. Casavant et al. designed a Microfluid Cell Concentrator (MCC) suitable for various cancer cell lines, as shown in Figure 10D. Compared with ELISPOT, MCC had comparable levels of CTCs isolated from blood samples of 5 patients [136]. Compared to the negative enrichment devices mentioned above, this method is simple and easy to operate. Lee et al. presented an integrated microfluidic chip called the μ-MixMACS chip that utilizes serpentine microchannels to generate Dean vortexes to enhance cell–channel interactions, as shown in Figure 10E. The chip has the ability to isolate CTCs from five blood samples, with 1–3 CTCs detected in each sample [67].

## 6. Microfluidic Technologies for Isolating Single CTCs

Multiple microfluidic platforms exist for enrichment of CTCs, but most of these methods only collect a subpopulation of CTCs. Because of the heterogeneity of cells in tissue and culture samples, molecular analyses using the entire cell population can only provide average data, rather than revealing important information about single cell in a subpopulation of cells, and therefore may mask important cell types or variants [137,138]. Cancer develops through a series of genetic mutations and cell selection, and DNA sequencing and genetic analysis from large cell populations is difficult even in a single tumor. Mutation information obtained from single cell in a cell subpopulation is a better predictor of cancer evolution and metastasis than the average information obtained from cell subpopulations, such as stem cells, cancer cells, and so on [139,140,141]. Genomic analysis of single cell is of great importance in cancer research, not only for predicting cancer progression and patient outcomes, but also for developing new drugs and providing new ideas for immunological studies [142,143,144]. Especially in the context of billions of normal blood cells, achieving genomic analysis of single cancer cell has an increasing clinical impact on cancer prognosis and treatment [19].

**Figure 11:**
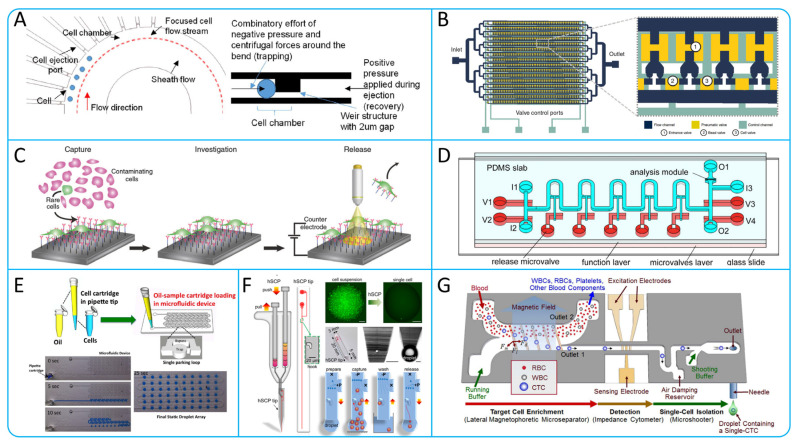
(**A**) Schematic illustration of microfluidic single-cell capture principle; reproduced from Reference [146], with permission from *Scientific Reports*. (**B**) Single-cell RNA sequencing of CTCs using Hydro-Seq.; reproduced from Reference [147], with permission from *Nature Communications*. (**C**) Schematic illustration of the working principle of capturing and releasing single CTCs based on the photoelectrochemical platform; reproduced from Reference [148], with permission from *Nature Communications*. (**D**) Schematic of an integrated microvalve chip capable of capturing at the single-cell level; reproduced from Reference [149], with permission from *Talanta*. (**E**) Schematic illustration of pipette working principle based on microfluidic cell isolation technology; reproduced from Reference [150], with permission from *Scientific Reports*. (**F**) Working principle of hand-held and integrated single-cell pipettes; reproduced from Reference [151], with permission from the *Journal of the American Chemical Society*. (**G**) Single-cell isolation device based on lateral magnetophoretic isolation and microfluidic dispensing; reproduced from Reference [152], with permission from *Analytical Chemistry*.

The traditional methods for single-cell genetic analysis include fluorescence in situ hybridization and PCR technology, but these two methods are limited by low sensitivity and throughput [145]. Currently, a variety of devices have been developed for the isolation of single CTCs. Yeo et al. have designed a microfluidic device (Figure 11A), which contains 10 cell chambers that utilize hydrodynamics to hold the cells within the chambers. The weir structure of the chambers increases fluid resistance, allowing other cells to be guided to the next chamber, achieving high purity (100%) isolation of single CTCs [146]. In order to overcome the scarcity of CTCs and the interference caused by the large number of blood cells, and thus to perform better molecular analysis of CTCs, Cheng et al. designed a Hydro-Seq chip, using barcoded beads. The structure of the chip is illustrated in Figure 11B. The chip contains 16 parallel channels with 50 cell chambers in each channel. By setting up cell valves, single CTCs can be captured into the chambers and RNA sequenced using barcode beads [147]. Parker et al. proposed a new capture and release platform that uses antibody modification and electrochemically cleavable technology. As shown in Figure 11C, the surface of this platform was modified with specific antibodies and an electrochemically cleavable linker [148]. On the other hand, Wang et al. designed a microfluidic chip capable of isolating single CTCs with high purity as shown in Figure 11D. The chip consists of three layers, where the single-cell functional layer contains 10 chambers for trap and release. The average recovery and purity of the chip for lung cancer cells was 92.5% and 94%, respectively [149].

In recent years, drop-based microfluidic technology has been rapidly developed. Bithi et al. proposed the pipette integrated microfluidic cell isolation technology as shown in Figure 11E. CTCs can be collected and detected without loss, and drug assayed [150]. Zhang et al. develop a hand-held single-cell pipet (hSCP) as shown in Figure 11F, which can isolate single cell suspensions directly from blood samples. The pipetting technique has the advantages of simple operation, rapidity and efficiency [151]. Most of the single-CTC isolation methods described above require pre-treatment to dilute the blood sample, which can lead to low-throughput results. Kim et al. designed a microfluidic chip that allows for high-purity, non-invasive isolation of single CTCs. As shown in Figure 11G, the chip uses a lateral magnetophoretic micro separator to isolate CTCs from blood samples, and then isolates single CTC by an electrical impedance cytometer and a single-cell micro shooter. In 200 μL of whole blood containing 20 CTCs, the isolation efficiency of 82.4% can be achieved [152].

## 7. Specific Clinical Application of CTCs

CTCs play an important role in clinical applications, and their detection and analysis are mainly applied to three aspects of cancer: early cancer diagnosis [153], cancer treatment response [154,155], and prognosis assessment [156]. Firstly, the detection of CTCs can be used for early cancer diagnosis and screening. Detecting the presence of tumor cells through blood samples is expected to detect tumors at an early stage and improve the success rate of treatment and survival rate [157]. Secondly, the number and characteristics of CTCs can be used as a monitoring indicator for the effectiveness of tumor treatment, which can be used to timely assess the efficacy of treatment and adjust the treatment plan [158]. Finally, the detection and analysis of CTCs can also be used to assess the prognosis of patients. High CTC levels are usually associated with poor prognosis and can be used as one of the important indicators for prognostic assessment, providing clinicians with a better basis for predicting and managing patients [159].

Currently, the clinical application of CTCs counting in a variety of solid cancers has been widely studied, including but not limited to cancer of the lung [158], prostate [160], breast [161], colorectum [162], and head and neck [163]. For example, CA199, as a standard biomarker for pancreatic cancer, is inaccurate in early diagnosis, while CTC counting with high sensitivity and specificity can make up for the deficiency of CA199. A dual-marker panel consisting of CA199 and CTC counts can significantly improve the performance of diagnosing PDAC [164]. Detailed clinical results were applied, as shown in Table 3.

Due to the short survival time of CTCs in blood, rapid and efficient methods are needed to isolate them to ensure their viability. In addition, in order to obtain a sufficient number of CTCs samples, high-throughput isolation methods need to be developed. Therefore, an ideal CTC isolation technique should have the following characteristics: efficient recovery, high purity, the ability to maintain cellular activity, and high-throughput isolation capability, which will help to analyze CTCs more accurately and provide a reliable database for relevant research and clinical applications.

## 8. Conclusions and Future Perspective

CTCs are cancer cells that are shed from the primary tumor and enter the circulation. The study of CTCs is important for cancer diagnosis, treatment, and prognosis judgement. By detecting the number and characteristics of CTCs, early cancer screening, personalized treatment and monitoring of treatment effects can be achieved.

In recent years, microfluidic system-based technologies have been extensively investigated for the isolation of CTCs for high-throughput capture and isolation. However, methods for isolating CTCs based on their physical and biological principles each have their own strengths and limitations, and cannot perfectly meet the needs of high recovery, high purity, and high throughput. Positive isolation methods based on the immunological properties of CTCs allow for high-purity enrichment of CTCs based on epithelial surface markers (EpCAM, EGFR, HER2), cytokeratins (CK8, CK18, and CK19), etc. However, CTCs are heterogeneous and can be EMT-transformed, which makes CTCs enrichment limited. In addition, the binding of antibodies to antigens affects the release of CTCs, leading to difficulties in downstream analyses. In contrast, negative isolation methods based on immunoaffinity to achieve CTCs enrichment, while avoiding some of the limitations, do not completely exclude other blood components, which tends to reduce purity and increase the difficulty of subsequent analyses. In addition, the density gradient isolation method and microfiltration membrane technologies, may cause damage to the CTCs integrity. Methods based on physical properties have low specificity, resulting in lower purity and recovery rate of enriched cells. For example, cell size-based microfiltration structures capture CTCs efficiently, but CTCs overlap in size with WBCs, resulting in lower purity of collected CTCs. Microfluidic devices that utilize the principles of DFF and inertial focusing have the advantage of simple operation and high throughput, but require the use of sheath fluid, which can dilute the concentration of CTCs. DEP methods based on the size and dielectric properties of CTCs can improve the purity of CTCs but are complex and costly. Combining physical and immunological principles can fully take advantage of their respective strengths and compensate for limitations. For example, high purity CTCs can be enriched under high-throughput conditions by combining microfiltration structures with immunomagnetic beads [128], DLD array with immunomagnetic capture [134,167], and acoustophoresis with immunomagnetic capture [130]. Label-free physical isolation and negative immunoaffinity methods are suitable for CTCs enumeration with high recoveries, whereas positive immunoaffinity methods are suitable for high-purity molecular analysis. Single-cell analysis provides more detailed information about single cells, revealing the cellular heterogeneity of the many different cell types in the same tumor. This understanding of cellular heterogeneity is critical for gaining deeper insights into tumor development and treatment response. Many microfluidic devices for single cell collection have been developed, such as droplet technology [151] and a photoelectrochemical platform [148] for releasing single CTC. Future single-cell isolation technologies should need to be combined with single-cell analysis technologies to reduce the risk of CTCs damage.

Isolation techniques for CTCs show great potential as a key tool in the field of cancer diagnosis and therapy but are still technically limited in clinical applications. Future trends suggest that combining methods based on physical and biological principles of CTCs will become the main direction. Methods based on physical principles are characterized by high throughput, while those based on biological principles have high specificity. The combination of these two principles will significantly improve the enrichment efficiency and purity of CTCs. Despite the challenges of downstream analysis of CTCs, with the continuous innovation of nanotechnology and microfluidics, we predict that CTCs isolation techniques will become more efficient, rapid, and economical, offering more possibilities for clinical applications. This development trend is expected to promote personalized cancer diagnosis and treatment, providing more effective medical solutions for patients, and promoting progress in the medical field.

## Figures and Tables

**Table 3:** Clinical applications of CTCs in cancer.

Clinical Applications	Methods	Cancer Type	Result Description	Reference
Early cancer detection	NE-imFISH and immunostaining	PDAC	When the cut-off value was 2 CTCs/3.2 mL, the ACU was 0.85, the specificity was 94%, and the sensitivity was 76%.	[164]
	CytoSorter^®^ microfluidic platform	BC	When the cut-off value was 2 CTCs/4 mL, the ACU was 0.86, the specificity was 95.4%, and the sensitivity was 76.56%.	[153]
	SE i-FISH	NSCLC	Sensitivity was 77.8 percent and specificity was 90 percent when CTCs (cut-off value 12 units) were combined with carcinoembryonic antigen (1.78 ng/mL)	[157]
Therapeutic response monitoring	RT-qPCR and Immunofluorescence staining	CRC	Patients whose NYONE^®^ test results were CTC-negative before surgery or whose CK20 mRNA relative expression was below the threshold after PCR analysis were tested for CTC immediately after surgery, and the results showed a significant increase.	[165]
	Flow cytometry and EasySep	Early-staged lung cancer	When the cut-off value was greater than 3 CTCs/mL, the number of CTCs decreased after surgery. The increased number of CTCs was positively correlated with cancer recurrence	[158]
Prognosis evaluation	Cell Search	MBC	progression-free survival: HR 1.79; overall survival: HR 2.72	[154]
	FISH and immunomagnetic enrichment	SCLC	The number of CTCs was correlated with lymph node metastasis (N), distant metastasis (M), TNM and NSE. High CTCS predicted poor prognosis, and the ROC curve AUC was always greater than 0.5.	[166]
	Cell Search	CRC	Multivariate analysis showed that only advanced age and preoperative CTCs detection were independent predictors of adverse OS. When the ratio is greater than 1 CTCs/7.5 mL, the overall survival and progression-free survival are shorter.	[162]

## Data Availability

No data were generated or used during the study.
